# Mental Health Help-Seeking Intentions and Perceived Barriers Among Filipino American Older Adults: An Exploratory COM-B-Informed Cross-Sectional Study

**DOI:** 10.3390/healthcare14152361

**Published:** 2026-08-03

**Authors:** Andrew Thomas Reyes, Reimund Serafica, Franz Henryk Vergara, Marlon Garzo Saria, Carol Manilay-Robles, Erwin William E. Leyva, Lorraine S. Evangelista

**Affiliations:** 1 School of Nursing, University of Nevada, Las Vegas, Las Vegas, NV 89154, USA; reimund.serafica@unlv.edu; 2 MedStar Harbor Hospital, Baltimore, MD 21225, USA; 3 Providence Saint John’s Health Center, Saint John’s Cancer Institute, Santa Monica, CA 90404, USA; marlon.saria@outlook.com; 4 Arleigh Burke Pavilion, McLean, VA 22101, USA; cmanilayrobles@yahoo.com; 5 College of Nursing, Pharmacy, and Allied Health Sciences, Negros Oriental State University, Dumaguete City 6200, Philippines; erwin.leyva@norsu.edu.ph; 6 Sue & Bill Gross School of Nursing, University of California, Irvine, Irvine, CA 92697, USA; evangell@hs.uci.edu

**Keywords:** Filipino Americans, older adults, mental health help-seeking, barriers, social support, COM-B, cultural factors, cultural beliefs, mental health stigma

## Abstract

**Highlights:**

**What are the main findings?**

- Filipino American older adults reported high health insurance coverage and social support, but only moderate intentions to seek professional mental health care.
- Stigma/interpersonal concerns were the most prominent perceived barriers to help-seeking for mental health problems and were associated with higher symptoms of depression and anxiety.

**What are the implications of the main findings?**

- Improving access to healthcare alone may be insufficient to increase mental health service utilization among Filipino American older adults.
- Culturally responsive interventions addressing stigma, mental health literacy, and community-based support may improve engagement with mental health services.

**Abstract:**

**Background/Objectives**: Filipino Americans are one of the least likely Asian American subgroups to use formal mental health services, even when mental health needs are present. This study used the Capability–Opportunity–Motivation–Behavior (COM-B) framework to examine mental health symptomatology, perceived social support, help-seeking intentions, and perceived barriers to mental health treatment among Filipino American older adults residing in the United States. **Methods**: We used a cross-sectional descriptive correlational design. A sample of 100 Filipino American older adults completed the Patient Health Questionnaire-9, Generalized Anxiety Disorder-7, an 8-item subset of the Multidimensional Scale of Perceived Social Support, the Mental Health Seeking Intention Scale, and the Perceived Barriers to Psychological Treatment scale. We performed descriptive statistics and correlation analyses using Pearson’s and Spearman’s coefficients. **Results**: Participants experienced low levels of depressive symptoms (*M* = 2.73, *SD* = 4.80) and anxiety symptoms (*M* = 2.56, *SD* = 4.11), high perceived social support (*M* = 5.32, *SD* = 1.83), moderate help-seeking intentions (*M* = 4.90, *SD* = 2.18), low overall perceived barriers to care (*M* = 1.67, *SD* = 0.90), and nearly universal health insurance coverage. Of the barrier domains, stigma/interpersonal barriers had the highest mean scores. Greater depressive and anxiety symptom severity was associated with higher overall perceived barriers to mental health care (*r* = 0.45–0.47) and with higher structural, motivational, and stigma/interpersonal barriers (*r* = 0.41–0.46). **Conclusions**: Despite high healthcare access and strong perceived social support, Filipino American older adults expressed only moderate intentions to seek professional mental health care. Culturally mediated factors may shape mental health help-seeking beyond structural barriers to access. The COM-B framework offers a useful lens for understanding help-seeking behavior and guiding culturally responsive interventions with Filipino American older adults.

## 1. Introduction

Mental health help-seeking behaviors have become a growing public health concern among older adults. Older adults frequently experience psychological distress associated with chronic illness, bereavement, loneliness, caregiving duties, and functional decline. Yet, they remain less likely than younger adults to seek formal mental health services [1]. A significant number of older adults perceive emotional distress as a normal part of aging, prefer to manage their difficulties independently, and rely on family members and informal networks rather than professional services [1,2]. Additional obstacles include social stigma, limited awareness of mental health issues, distrust of healthcare providers, transportation difficulties, and fear of becoming a burden to others [1,3]. Additional obstacles include social stigma, limited awareness of mental health issues, distrust of healthcare providers, transportation difficulties, and fear of becoming a burden to others [1,3]. Among older women, mental health help-seeking may also be shaped by gendered health experiences, including menopause-related physical and psychological changes [4] and exposure to gender-based violence [5], both of which may be associated with emotional distress, healthcare engagement, and willingness to seek professional support.

In the United States, Asian Americans aged 65 years and older constitute about 4.6% of the total older adult population, and this proportion is projected to increase to 8% by 2060 [6]. Among Asian Americans, mental health help-seeking is further complicated by cultural stigma, collectivist orientations, norms of emotional suppression, and concerns of losing face [7,8]. As a result, Asian Americans have a consistently lower use of mental health services compared to non-Hispanic Whites, even when mental health needs are significant [7,9]. Filipino Americans, in particular, have been identified as among the least likely subgroup within the Asian American population to seek formal mental health care [8,10]. Professional mental health treatment is frequently regarded as a measure of last resort, with individuals preferring first to seek support from family, friends, religious figures, and community networks [10]. The underutilization of mental health services among Filipino Americans is shaped by a range of sociocultural factors, including stigma, shame (“hiya”), concerns over family honor, emotional restraint, and a strong emphasis on self-sufficiency [8,10,11]. Cultural values such as “kapwa” (a sense of shared identity), the prioritization of interpersonal harmony, and indirect communication styles may further shape how emotional distress is understood and addressed [8]. In addition, colonial mentality has been associated with more negative attitudes toward professional mental health help-seeking in this population [12].

Despite a growing body of research on mental health service utilization among Asian Americans, the literature specifically addressing older Filipino Americans remains limited. Prior studies have often combined Asian American subgroups or focused primarily on younger populations [1,7]. Although existing research suggests that older Filipino Americans experience psychological distress while underutilizing professional mental health services [9], less is known about the factors that may explain mental health help-seeking in this population, including help-seeking intentions, perceived barriers to care, and perceived social support. A better understanding of these factors may help identify culturally relevant targets for improving mental health engagement among Filipino American older adults.

The present study was informed by the COM-B framework of Michie et al. [13], an element of the Behavior Change Wheel model. The COM-B framework posits that capability, opportunity, and motivation interact to explain behavior. According to Michie et al. [13], capability is an individual’s psychological and physical potential to perform a behavior; opportunity is the environmental and social factors that enable or deter someone from performing a behavior; and motivation is the reflective and automatic processes that energize and guide behavior. Mental health help-seeking may be explained not only by access to health care and mental health literacy, but also by sociocultural factors, including stigma, shame (“hiya”), emotional restraint, family-centered coping, and social norms related to mental illness. Accordingly, the COM-B framework was used in this study as a conceptual and interpretive framework for organizing factors that may explain mental health help-seeking among Filipino American older adults. Depressive and anxiety symptoms and recognition of emotional distress were conceptually aligned with capability; perceived social support, health insurance coverage, and structural barriers were considered indicators of opportunity; and help-seeking intentions, motivational barriers, and stigma/interpersonal barriers were reflective of motivation. Consistent with the exploratory nature of the study, COM-B constructs were not directly measured or tested. Rather, the framework was used as an interpretive lens to understand the study’s findings and identify potential behavioral and contextual factors relevant to mental health help-seeking among Filipino American older adults.

Based on these considerations, the purpose of this study was to examine mental health help-seeking intentions, perceived barriers to mental health care, perceived social support, depressive symptoms, and anxiety symptoms among Filipino American older adults. Specifically, the study sought to describe these factors and explore the relationships among psychological distress, perceived social support, perceived barriers to care, and mental health help-seeking intentions in this understudied population.

## 2. Materials and Methods

### 2.1. Study Design and Setting

This study employed a cross-sectional descriptive correlational design to examine mental health symptoms, perceived social support, mental health help-seeking intentions, and perceived barriers to mental health care among Filipino American older adults residing in the United States. The reporting of this study was guided by the Strengthening the Reporting of Observational Studies in Epidemiology (STROBE) Statement for cross-sectional studies.

### 2.2. Participants and Recruitment

Filipino American older adults were recruited from community-based settings across the United States through Filipino community events, social networks, community organizations, and electronic recruitment flyers containing a QR code linked to the study survey. Members of the Philippine Nurses Association of America (PNAA) assisted with community-based recruitment efforts.

Because participation was voluntary and recruitment occurred through multiple community channels, the number of individuals exposed to recruitment materials, assessed for eligibility, or who declined participation was not systematically tracked.

Eligible participants were Filipino or Filipino American adults aged 65 years or older, residing in the United States, and able to complete the survey in English. Individuals were excluded if they were younger than 65 years of age, did not identify as Filipino or Filipino American, resided outside the United States, or were unable to complete the survey in English.

The sample size was based on feasibility and participant availability during the study period. Because the study was exploratory in nature, an a priori power analysis was not performed. Recruitment continued until the available study period and community outreach efforts were completed, yielding 100 valid responses for analysis.

### 2.3. Measures

#### 2.3.1. Demographic Questionnaire

Participants completed a demographic questionnaire assessing age, gender, marital status, country of birth, years residing in the United States, educational attainment, employment status, living arrangement, household income, and health insurance coverage.

#### 2.3.2. Depressive Symptoms

Depressive symptoms were assessed using the 9-item Patient Health Questionnaire (PHQ-9), which measures depressive symptom severity during the previous two weeks [14]. Scores range from 0 to 27, with higher scores indicating greater depressive symptoms. The PHQ-9 has demonstrated strong reliability (Cronbach’s α = 0.86–0.89) and good validity in clinical and community populations [14]. In the current sample, the PHQ-9 demonstrated excellent internal consistency reliability (Cronbach’s α = 0.94).

#### 2.3.3. Anxiety Symptoms

Anxiety symptoms were measured using the 7-item Generalized Anxiety Disorder scale (GAD-7), which assesses generalized anxiety symptoms over the previous two weeks [15]. Scores range from 0 to 21, with higher scores indicating greater anxiety severity. The GAD-7 has demonstrated excellent reliability (Cronbach’s α = 0.92) and strong validity across clinical and research settings [15]. In the current sample, the GAD-7 demonstrated excellent internal consistency reliability (Cronbach’s α = 0.95).

#### 2.3.4. Perceived Social Support

Perceived social support was assessed using an 8-item subset of items from the Multidimensional Scale of Perceived Social Support (MSPSS) [16]. The original MSPSS is a 12-item instrument comprising three subscales assessing support from significant others, family, and friends. Participants rated each item on a 7-point Likert scale ranging from 1 (very strongly disagree) to 7 (very strongly agree). Scores were calculated by averaging responses across the retained items, with higher scores indicating greater perceived social support. Because only a subset of MSPSS items was administered, the resulting score should be interpreted as a global indicator of perceived social support rather than as a validated measure of the original MSPSS subscale structure. No exploratory factor analysis (EFA) or confirmatory factor analysis (CFA) was conducted to evaluate the factor structure of the administered item set. Therefore, the psychometric properties of the original 12-item MSPSS and its three-factor structure cannot be assumed to apply to the 8-item subset used in this study. The original MSPSS demonstrated good internal consistency reliability for the total scale (Cronbach’s α = 0.88) and strong reliability for the Significant Other (α = 0.91), Family (α = 0.87), and Friends (α = 0.85) subscales [16]. In the present sample, the 8-item MSPSS demonstrated excellent internal consistency (Cronbach’s α = 0.97). While this estimate suggests strong internal consistency among the items, very high reliability coefficients may also reflect substantial overlap or redundancy in items’ content and should therefore be interpreted with caution.

#### 2.3.5. Mental Health Help-Seeking Intention

Mental health help-seeking intention was measured using the 3-item Mental Help-Seeking Intention Scale (MHSIS), which assesses intention and willingness to seek professional mental health services [17]. Higher scores indicate greater help-seeking intention. The MHSIS has demonstrated excellent reliability (Cronbach’s α = 0.94) and evidence of predictive validity [17]. In the current sample, the MHSIS demonstrated excellent internal consistency (Cronbach’s α = 0.99). Although reliability coefficients approaching 1.00 may suggest substantial overlap among items, the MHSIS is intentionally designed as a brief, unidimensional measure of mental health help-seeking intention. The three items assess closely related expressions of intention (i.e., intending, planning, and trying to seek professional mental health care), which suggest a high degree of inter-item consistency observed in this sample. Therefore, the reliability estimate should be interpreted in the context of the scale’s brief, focused design.

#### 2.3.6. Perceived Barriers to Mental Health Care

Perceived barriers to mental health care were assessed using the 27-item Perceived Barriers to Psychological Treatment (PBPT) scale, which evaluates structural, attitudinal, and stigma-related barriers to seeking mental health services [18]. Higher scores indicate greater perceived barriers to care. The PBPT has demonstrated strong internal consistency reliability (Cronbach’s α = 0.92) and good criterion validity [18]. In the current sample, the PBPT (total) demonstrated excellent internal consistency reliability (Cronbach’s α = 0.98).

### 2.4. Procedures

Participants completed a self-administered survey in either electronic or paper format. Participation was voluntary, and informed consent was obtained before survey completion. Survey responses were collected anonymously, and no directly identifiable information was linked to participant responses. The mode of survey completion was not systematically recorded; therefore, the number of questionnaires completed in paper versus electronic format could not be determined. The study was approved by the University of Nevada, Las Vegas Institutional Review Board (IRB protocol #UNLV-2025-201).

### 2.5. Data Analysis

Data were analyzed using SPSS version 29. Descriptive statistics, including frequencies, percentages, means, and standard deviations, were used to summarize demographic characteristics and study variables. Given the exploratory nature of the study, analyses were limited to descriptive and correlational procedures. Pearson product–moment correlations were conducted to examine associations among continuous study variables, including depressive symptoms, anxiety symptoms, perceived social support, mental health help-seeking intentions, perceived barriers to care, age, and years residing in the United States. Spearman rank-order correlations were used to examine associations involving ordinal demographic variables, including educational attainment and annual household income. Analyses were conducted using the available data for each variable, and no data imputation was performed. Statistical significance was set at *p* < 0.05. Because the study was exploratory and cross-sectional in nature, multivariable regression analyses were not performed. Accordingly, findings should be interpreted as associations rather than evidence of independent predictors or causal relationships. The COM-B framework was not directly measured or statistically tested; rather, it was used as a conceptual and interpretive framework to organize and contextualize study findings.

## 3. Results

### 3.1. Participant Characteristics

The final sample consisted of 100 Filipino American older adults with a mean age of 71.81 years (*SD* = 6.36) who had resided in the United States for an average of 37.56 years (*SD* = 13.65). Most participants identified as female, married, and retired, and most reported at least a bachelor’s degree as the highest educational attainment. Additional demographic characteristics are presented in Table 1.

### 3.2. Mental Health Symptoms

Overall levels of depressive and anxiety symptoms were low. The mean PHQ-9 depression score was 2.73 (*SD* = 4.80), and the mean GAD-7 anxiety score was 2.56 (*SD* = 4.11). Depression and anxiety scores were strongly positively correlated (*r* = 0.83, *p* < 0.001).

### 3.3. Perceived Social Support

Participants reported high levels of perceived social support overall (*M* = 5.32, *SD* = 1.83). Perceived support from family (*M* = 5.38, *SD* = 1.90), significant others (*M* = 5.35, *SD* = 1.97), and friends (*M* = 5.26, *SD* = 1.93) was similarly elevated.

### 3.4. Mental Health Help-Seeking Intentions

Participants reported above-midpoint levels of intentions to seek mental health help (*M* = 4.90, *SD* = 2.18 on a 1–7 scale).

### 3.5. Perceived Barriers to Mental Health Care

Participants reported relatively low overall perceived barriers to mental health care (*M* = 1.67, *SD* = 0.90). Among barrier subdomains, stigma/interpersonal barriers had the highest mean score (*M* = 1.77, *SD* = 1.03), followed by structural/logistical barriers (*M* = 1.65, *SD* = 0.87) and attitudinal/motivational barriers (*M* = 1.59, *SD* = 0.94).

### 3.6. Correlational Findings

Correlations among mental health symptoms, perceived social support, help-seeking intention, and perceived barriers are presented in Table 2. Higher depressive symptom severity was significantly associated with greater overall perceived barriers to mental health care (*r* = 0.45, *p* < 0.001), as well as greater structural (*r* = 0.43, *p* < 0.001), motivational (*r* = 0.44, *p* < 0.001), and stigma/interpersonal barriers (*r* = 0.41, *p* < 0.001). Similarly, anxiety symptom severity was significantly associated with greater overall perceived barriers (*r* = 0.47, *p* < 0.001), as well as greater structural (*r* = 0.45, *p* < 0.001), motivational (*r* = 0.46, *p* < 0.001), and stigma/interpersonal barriers (*r* = 0.44, *p* < 0.001).

Perceived social support was negatively associated with depressive symptoms (*r* = −0.25, *p* = 0.011), anxiety symptoms (*r* = −0.26, *p* = 0.009), and perceived barriers to care (*r* = −0.30, *p* = 0.002). Higher perceived social support was also positively associated with greater mental health help-seeking intention (*r* = 0.36, *p* < 0.001).

Mental health help-seeking intention was negatively associated with perceived barriers to care (*r* = −0.28, *p* = 0.005).

Several demographic characteristics were significantly associated with perceived barriers to mental health care and are therefore reported here because of their relevance to understanding variation in help-seeking experiences among Filipino American older adults. Higher educational attainment, annual household income, and longer residence in the United States were associated with lower overall perceived barriers and lower structural, motivational, and stigma-related barriers to mental health help-seeking. Specifically, longer residence in the United States was associated with lower total perceived barriers (*r* = −0.326, *p* < 0.001), structural barriers (*r* = −0.336, *p* < 0.001), motivational barriers (*r* = −0.346, *p* < 0.001), and stigma/interpersonal barriers (*r* = −0.251, *p* = 0.012). Using Spearman rank-order correlations, higher educational attainment was associated with lower total perceived barriers (*r_s_* = −0.300, *p* = 0.002), structural barriers (*r_s_* = −0.363, *p* < 0.001), motivational barriers (*r_s_* = −0.282, *p* = 0.004), and stigma/interpersonal barriers (*r_s_* = −0.212, *p* = 0.034). Higher annual household income was similarly associated with lower total perceived barriers (*r_s_* = −0.279, *p* = 0.005), structural barriers (*r*_s_ = −0.301, *p* = 0.002), motivational barriers (*r_s_* = −0.282, *p* = 0.004), and stigma/interpersonal barriers (*r_s_* = −0.214, *p* = 0.033).

## 4. Discussion

Our findings indicate that Filipino American older adults included in this study show high levels of health insurance coverage, strong perceived social support, and relatively low structural barriers to care. Additionally, in our findings about perceived barriers to mental health care, stigma/interpersonal barriers had the highest mean score compared to structural and attitudinal barriers. Results also show that the participants have only moderate intentions to seek professional mental health help. These findings suggest that simply having access to healthcare may not be enough to promote participation in mental health services among this population. Findings imply that interpersonal, cultural, and psychological factors may be relevant to mental health among Filipino older adults even in the presence of adequate health insurance coverage and access to healthcare services. These findings are consistent with previous studies indicating that Filipino Americans and other Asian populations often underuse formal mental health services in the face of significant mental health needs [7,9,10].

We also found that greater depressive and anxiety symptom severity was associated with higher perceived barriers to care, reflecting a potential double burden in which participants reporting greater psychological distress also tended to report greater perceived barriers to seeking mental health care. These findings are consistent with previous studies indicating older adults’ uncertainty about whether emotional symptoms warrant professional help and therefore have discomfort and hesitation to discuss mental health concerns with professional healthcare providers [3,19]. Among Filipino American populations, these results are also in line with Sorkin et al. [9], who reported that Filipino American older adults were more likely than non-Hispanic White older adults to report mental distress symptoms, yet demonstrated lower utilization of mental health services and prescription medication use. Our findings regarding moderate intentions to seek professional mental health care are also consistent with Tuliao [8] and Teo et al. [1], who suggested that emotional distress may be normalized as part of aging, caregiving, chronic illness, or everyday stress, further decreasing the perceived need for professional intervention. Overall, these results suggest that differences in mental health service use among Filipino American older adults may be associated not only with access-related obstacles but also with culturally mediated behavioral and interpersonal factors that may explain care utilization.

Another notable finding was that higher levels of educational attainment and income, and greater lifetime residence in the United States, were associated with lower perceived barriers to mental health care. Our findings are consistent with prior literature linking socioeconomic status, acculturation, English proficiency, and familiarity with Western healthcare systems to greater willingness to seek professional mental health services among Filipino Americans [7,20].

The findings should also be interpreted in the context of the study’s English-language eligibility requirement. Participants were required to complete the survey in English, which may have resulted in the underrepresentation of older Filipino immigrants with limited English proficiency. This subgroup may experience greater linguistic, cultural, structural, and mental health literacy barriers to help-seeking than those captured in the present sample. Consequently, the relatively low structural barriers, strong perceived social support, and moderate help-seeking intentions observed in this study may not fully reflect the experiences of all Filipino American older adults, particularly more recent immigrants and those with lower levels of English proficiency. Therefore, caution is warranted when generalizing these findings to the broader Filipino American older adult population.

To provide a conceptual context for the findings, we applied the Capability–Opportunity–Motivation–Behavior (COM-B) framework [13] as an interpretive framework. Because COM-B constructs were not directly measured in this study, the framework was used to organize and contextualize findings rather than to test relationships among COM-B domains and help-seeking behavior. Study data were conceptually mapped to the COM-B domains to facilitate interpretation of psychological, social, cultural, and environmental factors that may play a role in mental health help-seeking among Filipino American older adults. This application of the framework provides a useful lens for understanding potential factors that may explain help-seeking behavior and identifying culturally responsive implementation strategies. In Table 3, we summarize the interpretation of the study findings within the COM-B framework and particularly highlight the potential barriers and implementation strategies.

Within the capability domain of the framework, participants reported minimal to low levels of depressive and anxiety symptoms and moderate intentions to seek mental health help. From a COM-B perspective, these findings may be relevant to the capability domain, as mental health literacy and recognition of emotional distress may help explain whether individuals consider seeking professional support. However, these interpretations should be viewed as conceptual rather than direct assessments of capability, which was not measured in this study. Filipino older adults may express emotional distress through somatic symptoms, interpersonal concerns, or spiritual frameworks rather than through conventional psychiatric symptom language [7,8,20]. Consequently, standard measures of depression and anxiety may not fully capture psychological burden in this population. Additionally, cultural practices of emotional control and normalization of distress were associated with participants’ modest intentions to seek mental health help. These results also corroborate previous reports that have reported stigma, shame (*hiya*), emotional control, family privacy, and worries about social judgment by Filipino Americans [8,10,11]. Therefore, professional mental health care may be seen as a “last resort,” with people deciding to turn to family members, close friends, clergy, or informal social networks to fall back on for support before using formal mental health care [10].

Conceptualizing the results through the framework’s opportunity domain, the positive association between perceived social support and help-seeking intentions is conceptually consistent with the COM-B framework, in which social resources are considered important components of the opportunity domain that may facilitate engagement with mental health care. Despite strong social support and access to care (e.g., high levels of health insurance coverage), participants had only moderate intentions to seek help. In addition, greater perceived barriers to care were associated with lower mental health help-seeking intentions, suggesting that even relatively modest barriers may be relevant to willingness to seek professional support among Filipino American older adults. Although overall perceived barriers were generally low, stigma/interpersonal barriers exhibited the highest mean scores across all domains of perceived barriers to care. Thus, the findings are consistent with Tuazon et al. [12], who demonstrated that greater social support may explain more favorable attitudes toward help-seeking among Filipino Americans. Conversely, social support in collectivist societies can reduce perceived need for formal mental health services when emotional concerns are managed within family and community networks [1]. This interpretation is also consistent with the Filipino cultural value of kapwa (shared identity and interconnectedness), which emphasizes the interconnected nature of the self and others. Within this cultural context, emotional concerns may be addressed through family, community, and interpersonal relationships, potentially reinforcing preferences for informal sources of support before seeking professional mental health services.

Within the motivation domain of the COM-B framework, participants reported moderate intention to seek professional mental health care despite relatively high healthcare access and social support. Cultural norms emphasizing resilience, emotional regulation, self-sufficiency, and avoiding burdening others have been discussed in the literature as potentially relevant to mental health help-seeking among Filipino Americans. They may provide context for the moderate help-seeking intentions observed in this sample. In line with previous research, fears of shame, embarrassment, “loss of face,” and fear of negative judgment may also deter professional mental health help-seeking among Filipino Americans [8,11,12]. Together, within the COM-B framework, these findings suggest that stigma and interpersonal concerns (e.g., self-reliance, emotional restraint, and concerns about social judgment) may warrant consideration alongside structural access barriers when interpreting mental health help-seeking among Filipino American older adults. Because the study employed a cross-sectional correlational design, these observations should be interpreted as associations rather than evidence of independent predictors or causal relationships.

In sum, the findings imply that just increasing mental health access may not be enough to reduce mental health disparities among Filipino American older individuals. Culturally responsive approaches that address mental health literacy, stigma, and culturally mediated beliefs while fostering trust in mental health services may be needed to strengthen engagement with professional mental health care in this population.

Several strengths should be noted. First, this study addresses an understudied population by focusing on mental health help-seeking intentions and perceived barriers among Filipino American older adults, a group that remains underrepresented in the mental health literature. Second, the study examined multiple factors relevant to help-seeking, including depressive symptoms, anxiety symptoms, perceived social support, help-seeking intentions, and perceived barriers to care. Third, the use of the COM-B framework provided a culturally informed conceptual lens for organizing and interpreting behavioral, social, and contextual factors relevant to mental health help-seeking. Finally, the study contributes preliminary data that may inform future longitudinal, mixed-methods, and intervention research involving Filipino American older adults.

### 4.1. Implications for Practice, Mental Health Service Delivery, and Future Research

Our findings have significant implications for primary care and community-based mental healthcare for Filipino American older adults. The low reported levels of depressive and anxiety symptoms observed in our sample may reflect culturally mediated patterns in the recognition, description, and expression of psychological distress. Therefore, healthcare providers need to employ culturally responsive mental health screening approaches. Additionally, normalizing mental health conversations in primary care interactions may also reduce stigma and increase earlier recognition of emotional distress in Filipino American older adults. Providers may need to develop culturally specific knowledge and understand how cultural beliefs and values may shape readiness to seek mental health care. For example, cultural values shared among Filipinos, such as hiya (shame), emotional restraint, family-centered coping, and avoidance of burdening others, may be relevant to mental health help-seeking behaviors [8,10]; therefore, integrating these values and beliefs in primary care interactions is essential.

Future research should further explore culturally mediated mental health help-seeking behaviors among Filipino American older adults through longitudinal studies, qualitative and mixed-methods designs, and community participatory approaches. Future intervention studies may examine the effectiveness of culturally tailored psychoeducation, community-based outreach, and behavioral health models in primary care settings for improving mental health help-seeking among Filipino American older adults.

### 4.2. Limitations

There are several limitations to consider when interpreting the results of this study. First, the cross-sectional design precludes drawing any conclusions about proximal causal links among psychological distress, perceived barriers, social support, and intentions to seek help for mental health. In addition, because analyses were limited to bivariate correlations, the study was not designed to identify independent predictors of mental health help-seeking intentions or perceived barriers to care. Future studies with larger sample sizes and multivariable analytic approaches are needed to examine the relative contributions of multiple factors simultaneously. Additionally, the COM-B framework was used as an interpretive framework rather than a directly measured or tested behavioral model. Study variables were conceptually mapped to COM-B domains to facilitate interpretation; therefore, conclusions regarding capability, opportunity, and motivation should be viewed as exploratory and conceptual rather than empirical assessments of these constructs. Additionally, an a priori power analysis was not conducted because the study was exploratory, and the sample size was determined by feasibility and participant availability during the study period. Although the sample was sufficient to identify several statistically significant associations, the absence of a formal power analysis limits the ability to indicate whether the study was adequately powered to detect smaller effects. In addition, several perceived barrier subscales demonstrated very high intercorrelations. For example, motivational barriers and stigma/interpersonal barriers were highly correlated, suggesting conceptual overlap among these domains. Therefore, findings from individual barrier subscales should be interpreted with caution, as observed overlap may limit the distinctiveness of specific barrier dimensions in this sample. In addition, perceived social support was assessed using an 8-item subset of MSPSS items rather than the full 12-item instrument. Although the administered items demonstrated excellent internal consistency, the subset was not independently validated, and no factor analytic procedures were conducted to evaluate its dimensional structure. Furthermore, the very high internal consistency coefficient observed in this sample may reflect overlap or redundancy in the items’ content. Therefore, findings involving perceived social support should be interpreted with caution.

Second, the use of convenience sampling may limit the generalizability of the findings to the broader Filipino American older adult population in the United States. Participants were recruited through community-based settings, social networks, community organizations, electronic recruitment flyers, and PNAA-assisted outreach, which may have resulted in a sample of individuals who were more socially connected, more engaged with Filipino community networks, and more comfortable participating in health-related research. Because the denominator of eligible individuals reached through community and electronic recruitment could not be determined, the response rate and nonresponse bias could not be estimated. Participants were mostly female, well-educated, insured, and long-time U.S. residents, potentially excluding people of lower socioeconomic status, with less access to healthcare, and with more recent immigrant histories. Therefore, these limitations may under-represent more vulnerable or recently immigrated Filipino American older adults. As a result, the sample may not fully reflect the experiences of Filipino American older adults who are more socially isolated or less connected to community and healthcare systems. This potential selection bias should be considered when interpreting the findings. Furthermore, because all participants were born in the Philippines, the findings may primarily reflect the experiences of older Filipino immigrants residing in the United States. They may not fully represent the broader population of Filipino American older adults, which includes both immigrant and U.S.-born individuals.

Third, all the measures used self-report data and may be subject to recall bias, social desirability bias, and cultural predispositions toward emotionally restrained behavior or underreporting of psychological distress. In Filipino cultural contexts, emotional distress may also be described through somatic symptoms rather than being identified more explicitly as depression or anxiety. Because menopausal status, menopause-related symptoms, and lifetime exposure to gender-based violence were not assessed, future studies should examine how gendered health experiences may shape mental health help-seeking among older Filipino American women. In addition, because recruitment occurred through multiple community-based channels, detailed recruitment flow data (e.g., the number of individuals approached, screened, determined to be eligible, or who declined participation) were unavailable, limiting the ability to fully characterize participant flow through the study.

Fourth, participation was restricted to individuals who could complete the survey in English. As a result, older Filipino immigrants with limited English proficiency may have been excluded from the study. This is an important methodological limitation because individuals with limited English proficiency may experience greater linguistic, cultural, structural, and mental health literacy barriers to accessing mental health services and may therefore represent a particularly vulnerable subgroup. Consequently, the findings may not be generalizable to all Filipino American older adults, especially more recent immigrants and those with limited English proficiency. Future research should incorporate translated instruments and culturally and linguistically appropriate recruitment strategies to better capture the experiences of this population.

Despite these limitations, this study adds to the scarce body of research on culturally mediated help-seeking behaviors in mental health among Filipino American older adults. The study findings offer initial insight into behavioral, interpersonal, and contextual factors underlying engagement in mental health care in this population.

## 5. Conclusions

This study contributes to the limited literature on mental health service seeking among Filipino American older adults by demonstrating the complex interplay of psychological distress, cultural norms, perceived barriers, and help-seeking intentions. Despite reporting a high level of general access to care, health insurance, and perceived social support, mental health help-seeking intentions were moderate, indicating that access to care alone may not be sufficient to support engagement with mental health services in this population.

The findings suggest that social, behavioral, and cultural factors may be relevant to understanding mental health help-seeking among Filipino American older adults. The COM-B framework served as an interpretive lens for understanding the findings and may be useful for informing future research and culturally responsive interventions. Taken together, this study highlights the need to consider not only structural access to care, but also cultural, relational, and behavioral factors relevant to mental health help-seeking among Filipino American older adults. Future research using larger and more diverse samples should employ multivariable analytic approaches to examine potential demographic, cultural, health-related (including gender- and life-stage-related), and contextual factors associated with mental health help-seeking and perceived barriers to care among Filipino American older adults.

## Figures and Tables

**Table 1:** Demographic characteristics of Filipino American older adult participants.

Characteristic	Category	*n* (%) or Mean ± *SD*
Age	Mean Age in Years	71.81 ± 6.36
	Range of Age	65–93
Residence in the United States (U.S.)	Years residing in the U.S.	37.56 ± 13.65
	Range of years in the U.S.	10–66
Gender	Female	81 (81.0%)
	Male	19 (19.0%)
Country of Birth	Philippines	100 (100%)
Marital Status	Single	6 (6.0%)
	Married	60 (60.0%)
	Divorced/Separated	7 (7.0%)
	Widowed	27 (27.0%)
Current Living Arrangement	Lives alone	21 (21.0%)
	Lives with spouse or partner	34 (34.0%)
	Lives with other family members	23 (23.0%)
	Lives with spouse/partner and other family members	20 (20.0%)
	Lives with a non-family member	2 (2.0%)
Highest Educational Attainment	High school graduate	1 (1.0%)
	Some college	5 (5.0%)
	Associate/vocational degree	6 (6.0%)
	College graduate	15 (15.0%)
	Bachelor’s degree	38 (38.0%)
	Graduate/professional degree	35 (35.0%)
Employment Status	Employed	19 (19.0%)
	Unemployed	1 (1.0%)
	Homemaker	2 (2.0%)
	Retired	78 (78.0%)
Annual Household Income	Less than $24,999	7 (7.0%)
	$25,000–$49,999	10 (10.0%)
	$50,000–$74,999	20 (20.0%)
	$75,000–$99,999	18 (18.0%)
	Greater than $100,000	26 (26.0%)
	Prefer not to answer	19 (19.0%)
Health Insurance Coverage	Government insurance (Medicare/Medicaid) ^1^	57 (57.0%)
	Private/Commercial insurance	4 (4.0%)
	HMO ^2^	12 (12.0%)
	TriCare ^3^	1 (1.0%)
	Medicare/Medicaid + commercial supplement	19 (19.0%)
	Medicare/Medicaid + TriCare supplement	7 (7.0%)

^1^ Medicare and Medicaid are U.S. government-funded health insurance programs primarily for older adults, low-income individuals, and certain individuals with disabilities. ^2^ HMO = Health Maintenance Organization. ^3^ TriCare is a U.S. government health insurance program for military personnel, retirees, and their families. Percentages are based on valid responses; minor discrepancies may occur due to rounding.

**Table 2:** Correlations among mental health symptoms, social support, help-seeking intentions, and perceived barriers.

Variable	*M*	*SD*	1	2	3	4	5	6	7	8
1. Depression	2.73	4.80	—							
2. Anxiety	2.56	4.11	0.83 ***	—						
3. Social Support	5.32	1.83	−0.25 *	−0.26 **	—					
4. Help-Seeking Intention	4.90	2.18	−0.25 *	−0.20	0.36 ***	—				
5. Total Perceived Barriers	1.67	0.90	0.45 ***	0.47 ***	−0.30 **	−0.28 **	—			
6. Structural Barriers	1.65	0.87	0.43 ***	0.45 ***	−0.25 *	−0.28 **	0.94 ***	—		
7. Motivational Barriers	1.59	0.94	0.44 ***	0.46 ***	−0.31 **	−0.27 **	0.98 ***	0.89 ***	—	
8. Stigma/Interpersonal Barriers	1.77	1.03	0.41 ***	0.44 ***	−0.31 **	−0.25 *	0.95 ***	0.79 ***	0.94 ***	—

Note. Values represent Pearson correlation coefficients. *M* = mean; *SD* = standard deviation. * *p* < 0.05; ** *p* < 0.01; *** *p* < 0.001 (two-tailed).

**Table 3:** Conceptual mapping of study findings to the COM-B framework for mental health help-seeking among Filipino American older adults.

COM-B Component	Study Findings Among Filipino American Older Adults	Potential Barriers	Potential Implementation Strategies
Capability	Minimal-to-low depressive/anxiety symptoms; moderate help-seeking intentions	Potential gaps in mental health literacy; possible normalization or somatic interpretation of emotional distress; limited familiarity with psychotherapy and formal mental health services	Culturally tailored psychoeducation; mental health literacy programs; routine mental health screening and education in primary care
Opportunity	High insurance coverage and social support despite only moderate help-seeking intentions	Potential sociocultural and contextual influences, including stigma, shame (hiya), emotional restraint, family privacy norms, the Filipino value of shared identity and interconnectedness (kapwa), and preference for informal support networks	Integrated behavioral health in primary care; Filipino-centered community outreach; language-accessible services; partnerships with churches and Filipino organizations; stigma-reduction initiatives; increased availability of Filipino American mental health providers.
Motivation	Moderate help-seeking intentions despite high insurance coverage and strong perceived social support	Potential motivational influences, including self-reliance, preference for family-based coping, fear of judgment or “loss of face,” and avoidance of emotional disclosure	Motivational interviewing; culturally congruent framing of mental wellness; family-inclusive approaches; trauma-informed and culturally responsive care

Note. COM-B constructs were not directly measured. Findings were conceptually mapped to the COM-B domains to facilitate interpretation of behavioral, cultural, social, and contextual factors relevant to mental health help-seeking among Filipino American older adults. Potential barriers and implementation strategies are interpretive and informed by both study findings and prior literature.

## Data Availability

The data presented in this study are available on request from the corresponding author. The data are not publicly available due to privacy and ethical restrictions related to the protection of participant confidentiality.
